# Extensive Spontaneous Pneumomediastinum With Suspected Recurrence in a Young Woman: A Case of Hamman Syndrome

**DOI:** 10.7759/cureus.105861

**Published:** 2026-03-25

**Authors:** Amr Ahmed, Ramadan Ahmed, Ahmed Yusuf, Muhammad Bilal, Marwa Elsadig, Md Monjur Morshed

**Affiliations:** 1 Medicine, Lincoln County Hospital, United Lincolnshire Teaching Hospitals NHS Trust, Lincoln, GBR; 2 Otolaryngology, Lincoln County Hospital, United Lincolnshire Teaching Hospitals NHS Trust, Lincoln, GBR; 3 Geriatrics, Lincoln County Hospital, United Lincolnshire Teaching Hospitals NHS Trust, Lincoln, GBR

**Keywords:** boerhaave syndrome, emergency medicine, emphysema subcutaneous, hamman syndrome, macklin effect, recurrent pneumomediastinum, spontaneous pneumomediastinum (spm)

## Abstract

Hamman syndrome, also known as spontaneous pneumomediastinum (SPM), is a rare and often under-recognized condition characterized by the presence of free air within the mediastinum in the absence of trauma or iatrogenic causes, most commonly affecting young adults after events that increase intrathoracic pressure. We report a case of SPM in a previously healthy 19‑year‑old woman presenting with neck and chest pain after throat discomfort and repeated vomiting. Imaging confirmed extensive pneumomediastinum, and perforation was excluded. She was managed conservatively with complete recovery. This case highlights the importance of considering spontaneous pneumomediastinum in young patients presenting with neck or chest discomfort following episodes of increased intrathoracic pressure, as it can clinically mimic life‑threatening conditions such as esophageal rupture, mediastinitis, or tension pneumothorax, and underscores the role of prompt imaging in excluding life-threatening causes while guiding appropriate conservative management.

## Introduction

Spontaneous pneumomediastinum (SPM) is defined as the presence of free air within the mediastinum in the absence of trauma, invasive procedures, or mechanical ventilation [1,2]. First described by Louis Hamman in 1939, the condition is also referred to as Hamman syndrome [3], while Hamman’s sign refers to a crunching sound heard with the heartbeat.

The underlying mechanism is commonly explained by the Macklin effect, whereby increased intra-alveolar pressure leads to alveolar rupture, allowing air to dissect along bronchovascular sheaths into the mediastinum [1,4]. Although SPM is generally benign and self-limiting, it can mimic more serious conditions such as esophageal rupture or tension pneumothorax, necessitating careful evaluation [1,2].

SPM most frequently affects young adults, particularly males [5-7], and is often associated with precipitating factors including forceful vomiting, asthma exacerbations, coughing fits, intense physical exertion, or Valsalva maneuvers [1,5-7]. Given its rarity and nonspecific presentation, a high index of suspicion is required for diagnosis.

## Case presentation

A previously healthy 19-year-old woman presented to the emergency department with a two-day history of progressive throat discomfort and multiple episodes of non-bloody vomiting, followed later by the onset of bubbling sensations in the neck, mild chest tightness, and mild difficulty in swallowing. She denied fever, dyspnea, trauma, recent endoscopic procedures, or illicit drug use. She was a non-smoker with no significant past medical history. She reported a similar but milder episode one year prior that did not require medical evaluation.

On admission, her vital signs were within normal limits: a temperature of 36.8°C, heart rate of 92 bpm, blood pressure 118/72 mmHg, respiratory rate of 18 breaths/min, and oxygen saturation of 98% on room air; the Glasgow Coma Scale (GCS) score was 15/15. Palpation of the neck revealed crepitus consistent with subcutaneous emphysema. Cardiovascular and respiratory examinations were otherwise unremarkable. No Hamman’s crunch was clearly auscultated.

Initial laboratory investigations on admission revealed a significant leucocytosis with a white cell count of 20.3 × 10⁹/L, predominantly driven by neutrophilia (17.02 × 10⁹/L), as well as mild hemoconcentration (hemoglobin 154 g/L). Notably, the initial C-reactive protein (CRP) level was within normal limits at 4.9 mg/L, which does not suggest an established acute infectious process such as mediastinitis, although early infection cannot be excluded. Renal profile and liver function were largely preserved. Venous blood gas analysis demonstrated a mild respiratory alkalosis (pH 7.528, pCO_2_ 3.53 kPa), findings consistent with her clinical presentation of hyperventilation due to distress and recurrent emesis (Table 1).

**Table 1:** Test results demonstrating initial significant leucocytosis and hemoconcentration, which trended downward over the course of admission pCO_2_: partial pressure of carbon dioxide; eGFR: estimated glomerular filtration rate; ALT: alanine aminotransferase

Parameter	Reference Range	Day 0	Day 1	Day 3
White cell count (×10⁹/L)	4.3-11.2	20.3	11.2	9.0
Neutrophils (×10⁹/L)	2.1-7.4	17.02	8.89	6.79
Hemoglobin (g/L)	117-149	154	122	131
C-reactive protein (mg/L)	0-5	4.9	8.1	6.3
Potassium (mmol/L)	3.5-5.3	3.5	3.7	3.5
eGFR (EPI 2009) (mL/min)	90-200	>90	>90	>90
Bilirubin (umol/L)	0-21	24	21	12
ALT (U/L)	0-33	16	8	10
pH (venous)	7.320-7.430	7.528	-	-
pCO_2_ (venous) (kPa)	5.30-8.10	3.53	-	-
Actual bicarbonate (mmol/L)	22.0-29.0	22.0	-	-

**Figure 1:**
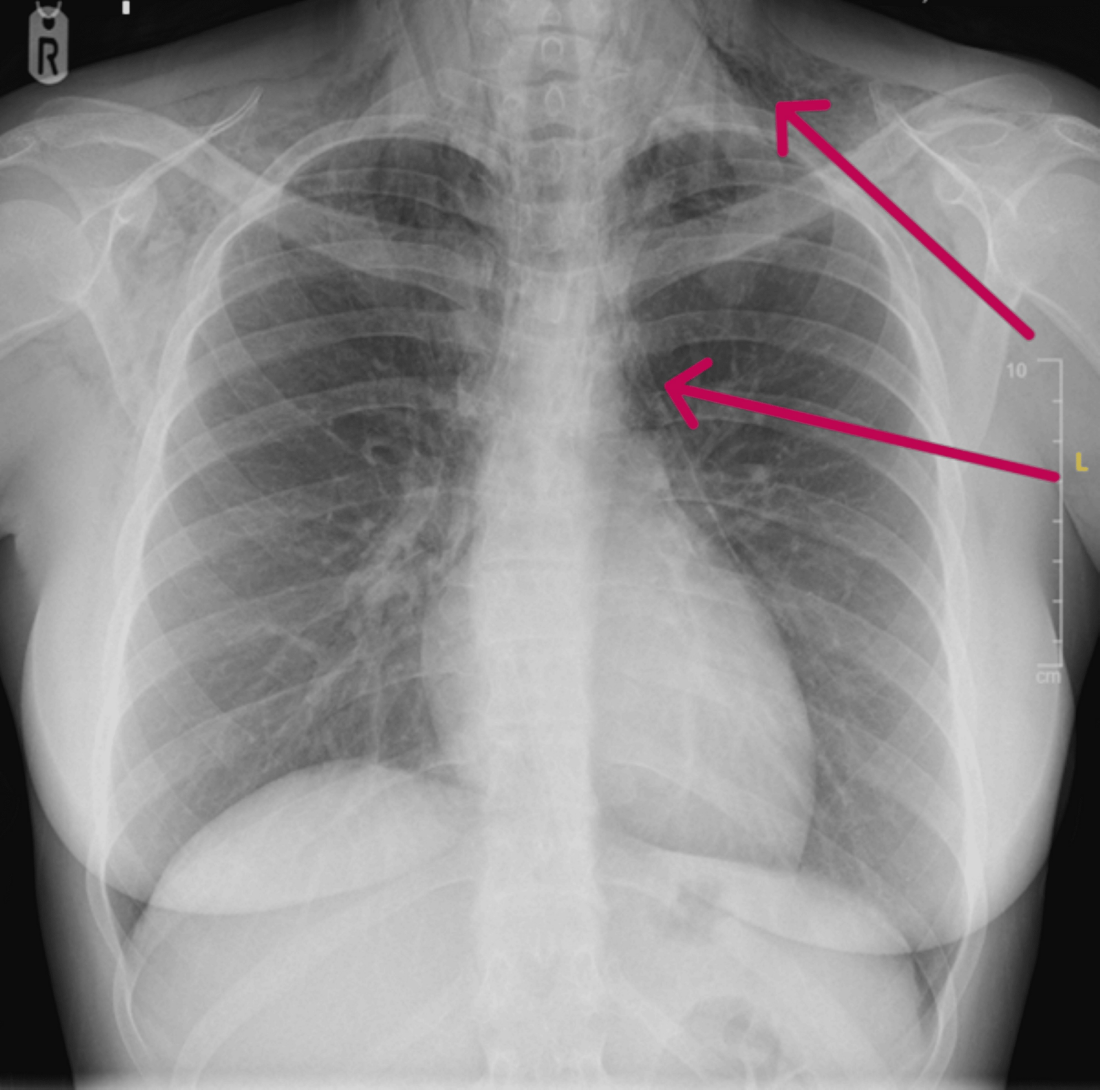
Chest radiograph showing pneumomediastinum and extensive subcutaneous emphysema seen across the soft tissues of the neck and upper chest as demonstrated by red arrows.

A chest radiograph (CXR) demonstrated extensive pneumomediastinum and subcutaneous emphysema (Figure 1).

**Figure 2:**
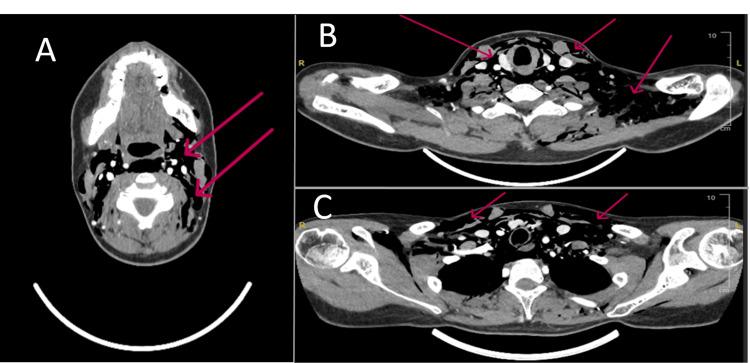
Computed tomography (CT) of the neck and upper chest highlighting the massive extent of the subcutaneous emphysema. (A-C) Sequential views demonstrating a large volume of subcutaneous air tracking from the skull base, inferior to the mastoids, through the cervical fascial planes and into the upper chest wall, as indicated by the red arrows.

**Figure 3:**
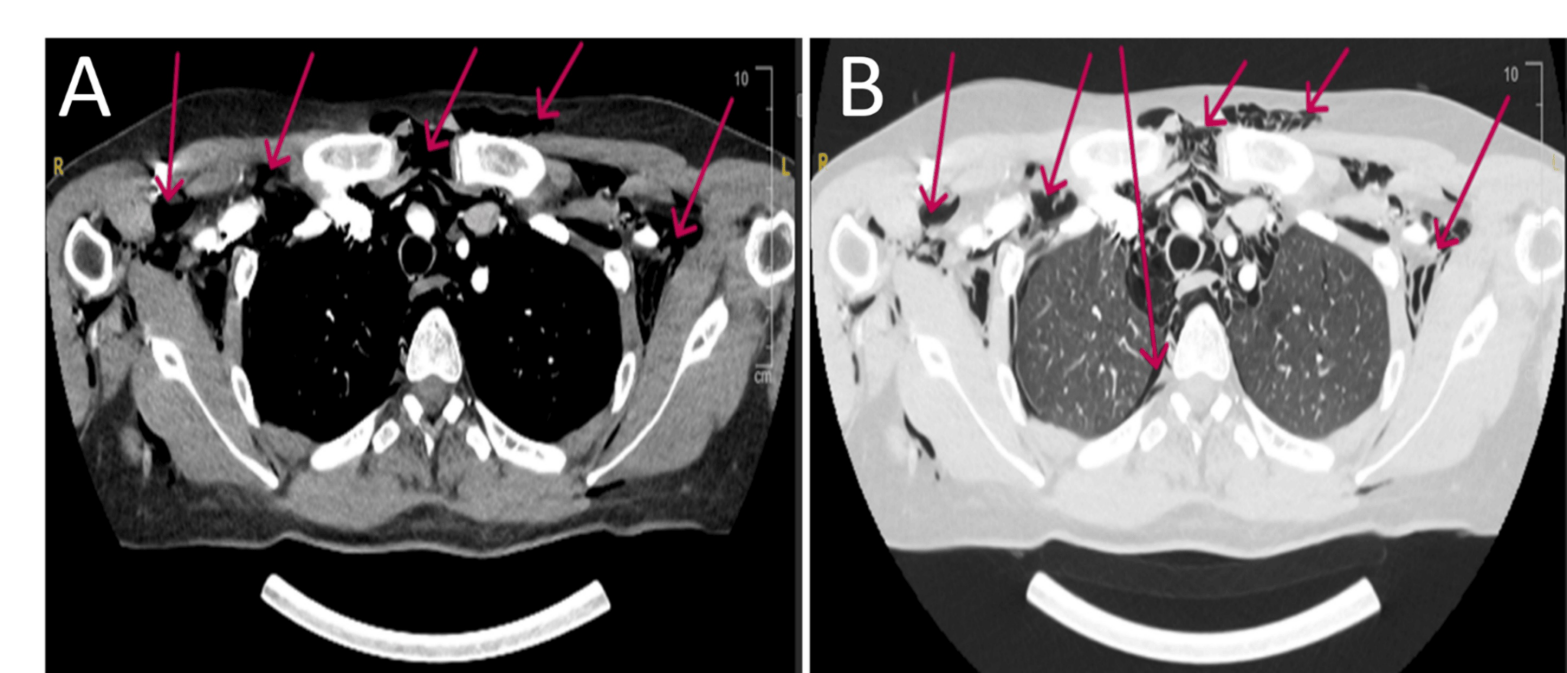
Computed tomography (CT) of the chest revealing substantial intrathoracic free air. (A) Mediastinal window showing a large volume of both subcutaneous and mediastinal air (red arrows). (B) Corresponding lung window further detailing the extensive subcutaneous and mediastinal emphysema (red arrows), alongside a small right-sided pneumothorax.

**Figure 4:**
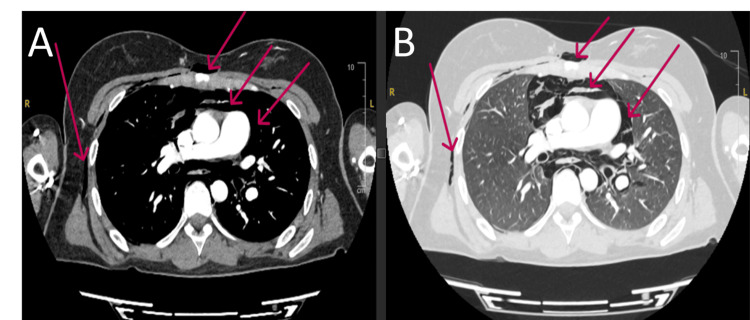
Axial computed tomography (CT) scan cross-sections at the level of the great vessels. (A) Mediastinal window showing significant pneumomediastinum and overlying subcutaneous air (red arrows). (B) Corresponding lung window further detailing the subcutaneous emphysema and mediastinal air dissecting around the cardiovascular structures (red arrows).

Subsequent CT imaging of the neck and chest demonstrated extensive pneumomediastinum with subcutaneous air tracking from the skull base through the cervical fascial planes to the anterior chest wall, along with a mild right-sided pneumothorax (Figures 2-4).

**Figure 5:**
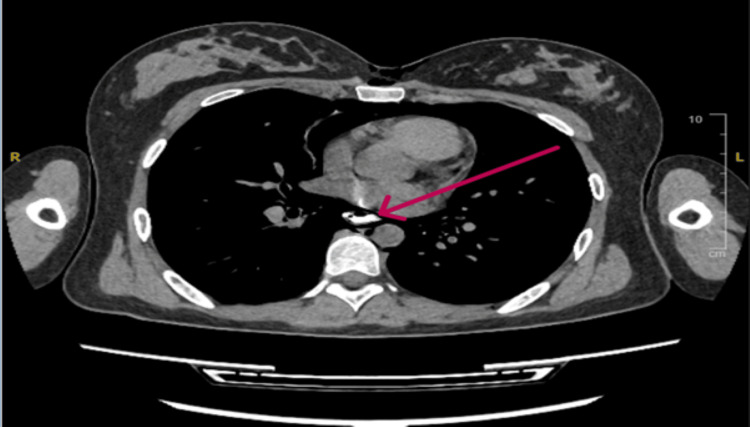
Axial CT of the chest with water-soluble oral contrast. The red arrow indicates contrast safely contained within the esophageal lumen. There is no evidence of extravasation, which argues strongly against esophageal or pharyngeal perforation (Boerhaave syndrome).

Given the patient's history of persistent vomiting and the high clinical suspicion for Boerhaave syndrome, a stat dose of intravenous piperacillin/tazobactam 4.5g was administered empirically for potential mediastinitis. Subsequently, a CT scan of the neck and chest with water-soluble oral contrast was obtained that demonstrated no evidence of contrast leak or esophageal or pharyngeal perforation (Figure 5).

Based on clinical stability, imaging findings, and exclusion of secondary causes, a diagnosis of spontaneous pneumomediastinum (Hamman syndrome) was made. The patient was admitted for close monitoring due to extensive subcutaneous air and large pneumomediastinum. Management included analgesia, antiemetics, and serial clinical assessments. She was kept nil per mouth until the CT scan with oral contrast excluded esophageal or pharyngeal perforation, after which oral intake was resumed. No supplemental oxygen, intravenous fluids, further antibiotics, or surgical intervention was required. The patient remained stable with gradual symptomatic improvement and was discharged home after three days with an outpatient phone consultation follow‑up in one month. During the telephonic follow-up, she reported complete clinical recovery. Throat discomfort resolved within one week, while the cervical ‘bubbling’ sensation subsided over the subsequent two to three weeks. A repeat chest CT scan without contrast performed three months later demonstrated complete resolution, with no evidence of pneumomediastinum or underlying emphysematous bullae. Only a couple of bilateral calcified granulomas were seen in both lungs, in keeping with previous infection.

## Discussion

Spontaneous pneumomediastinum is a rare clinical entity, with reported incidence rates in the emergency department varying between approximately 1 in 14,000 and 1 in 44,000 visits, in published series [6,7]. The pathophysiology is most commonly attributed to the Macklin effect, in which alveolar rupture secondary to increased intrathoracic pressure allows air to track centrally toward the mediastinum [1,4]. In this case, repetitive vomiting likely generated sufficient intrathoracic pressure to precipitate alveolar rupture [5,6]. SPM can occasionally involve other compartments; for instance, it may be associated with mild pneumoperitoneum via air dissection along fascial planes into the retroperitoneal space [1,2], or a concurrent pneumothorax, as seen in our patient, when mediastinal air ruptures through the mediastinal pleura.

The primary clinical challenge lies in differentiating benign SPM from life-threatening causes such as esophageal perforation (Boerhaave syndrome), tension pneumothorax, or necrotizing mediastinitis [1,8]. CT imaging plays a crucial role in diagnosis and in excluding secondary causes [1,2]. While routine esophagography is debated in stable patients without concerning features, contrast-enhanced imaging was warranted in this case due to the history of vomiting [8].

Management of uncomplicated SPM is typically conservative [2,7,9]. Oxygen therapy may accelerate reabsorption of free air [7,9]. Hospital admission is often recommended for observation, particularly when the diagnosis is uncertain, symptoms are significant, or secondary causes have not been definitively excluded. Prognosis is excellent, and recurrence is rare.

This case is noteworthy for several reasons. First, although SPM typically demonstrates a slight male predominance [5,6], our patient was a female non‑smoker, highlighting that SPM can also occur outside the typical demographic profile. Second, she experienced a similar episode one year earlier. Although her prior episode one year ago was not radiographically confirmed, the identical clinical presentation strongly suggests recurrence. Recurrence is uncommon, with most patients experiencing a single self-limited episode; literature suggests recurrence rates are only around 1%-2% [5,6]. Finally, complete resolution was achieved with conservative management alone, further supporting the generally favourable prognosis of uncomplicated SPM.

## Conclusions

Hamman syndrome is a rare but generally benign condition that should remain in the differential diagnosis of young patients presenting with chest or neck symptoms following episodes of increased intrathoracic pressure. Early recognition is crucial, and appropriate imaging is essential to exclude serious underlying pathologies such as esophageal rupture. In clinically stable patients without evidence of perforation, conservative management with observation is usually sufficient and associated with excellent outcomes.
